# Speciality preferences in Dutch medical students influenced by their anticipation on family responsibilities

**DOI:** 10.1007/s40037-014-0149-5

**Published:** 2014-11-14

**Authors:** Margret Alers, Tess Pepping, Hans Bor, Petra Verdonk, Katarina Hamberg, Antoine Lagro-Janssen

**Affiliations:** 1 Unit Gender and Women’s Health, Department of Primary and Community Care, Radboud University Medical Centre, ELG-117, PO Box 9101, 6500 HB Nijmegen, the Netherlands; 2 Department of Medical Humanities, School of Medical Sciences, EMGO Institute for Health and Care Research, VU University Medical Centre, Amsterdam, the Netherlands; 3 Department of Public Health and Clinical Medicine, Family Medicine, Umeå University, Umeå, Sweden

**Keywords:** Medical graduates, Speciality choices, Gender, Working hours, Work–life balance

## Abstract

Physician gender is associated with differences in the male-to-female ratio between specialities and with preferred working hours. We explored how graduating students’ sex or full-time or part-time preference influences their speciality choice, taking work-life issues into account. Graduating medical students at Radboud University Medical Centre, the Netherlands participated in a survey (2008–2012) on career considerations. Logistic regression tested the influence of sex or working hour preference on speciality choice and whether work-life issues mediate. Of the responding students (N = 1,050, response rate 83, 73.3 % women), men preferred full-time work, whereas women equally opted for part time. More men chose surgery, more women family medicine. A full-time preference was associated with a preference for surgery, internal medicine and neurology, a part-time preference with psychiatry and family medicine. Both male and female students anticipated that foremost the career of women will be negatively influenced by family life. A full-time preference was associated with an expectation of equality in career opportunities or with a less ambitious partner whose career would affect family life. This increased the likelihood of a choice for surgery and reduced the preference for family medicine among female students. Gender specifically plays an important role in female graduates’ speciality choice making, through considerations on career prospects and family responsibilities.

## Introduction

The feminization of the medical profession is proceeding rapidly and there are a number of medical specialities that can be designated in which the male-to-female ratio is disproportionate [1–3]. Studies amongst medical graduates also show that women make different speciality choices compared with their male counterparts [4, 5]. A variation in the extent of the gender differences in speciality choices may have a cross-cultural component [6, 7]. In general, women are under-represented in the surgical profession, and the number of male graduates entering the practice of obstetrics–gynaecology has significantly declined [8, 9].

The majority of physicians, across all specialities, work full time at their present job [1–3]. Working part time is difficult to achieve in some hospital specialities [10, 11]. Surgeons are least likely to work part time [12]. At present, medical specialists working part time are mostly female and have children below the age of five [13, 14]. Although working fewer hours could benefit physicians and patients, e.g. sustained attention and concentration [15], part-time work decreases career opportunities [16, 17]. At the same time, actual and preferred working hours differ [14]. Both male and female medical graduates express a declining interest in specialities with less controllable lifestyles due to the work-life balance [18]. Both have also expressed a preference for working part time in the future [10, 19]. When taking differences in the male-to-female ratio across specialities into account, the transformation of a full-time workforce to a part-time one may lead to a mismatch in the supply and demand of physicians.

Reasons for changing from a full-time workload to part time are work-life issues such as family responsibilities, for example childcare [20, 21]. Amongst female residents, work and time-related aspects were more important and career-related aspects were less important factors for speciality choice, compared with men [21]. The career paths of male and female physicians reflect gendered expectations on women being caretakers and men being breadwinners [22]. Because of their family life, women wish for a more controllable lifestyle and structured work schedule.

After a clerkship in which the student meets several different working cultures, a reliable endpoint can be found for the final choice for a speciality. For the majority of students, medical school has the potential to influence the final choice of speciality. Speciality preferences of female and male medical students may be reinforced or changed by the moment when they make their final speciality choice [23, 24]. Women may reject some specialities as they may believe the speciality does not allow part-time work, regardless of the accuracy of such a notion.

With our study, we aim to investigate how graduating medical students’ sex and full-time or part-time preference influences speciality choice and whether work-life issues play a part in this. More specifically, our study among graduating students aims to answer [1] what is the influence of sex or a full-time or part-time preference on their speciality choice, [2] what is the relation of sex or a full-time or part-time preference on work-life issues and [3] whether work-life issues mediate (a) the relationship between sex and speciality choice or (b) the relationship between full-time or part-time preference and speciality choice.

## Methods

### Participants

A cohort of graduating medical students from the Radboud University Medical Center, the Netherlands (N = 1,267, 70.1 % women) participated in a cross-sectional survey on career considerations between 2008 and 2012. With regard to medical ethical approval in the Netherlands, as Dutch legislation did not require ethical permission, we followed procedures as later described by the Ethics Review Board of the Netherlands Association for Medical Education (NVMO). This Review Board was not in place at the time the data were collected. Students were informed in advance of the survey that participation was voluntary and that data would be anonymized and treated confidentially. This study was part of the Gender Challenges in Medical Education Project [25].

### Measures

First, we collected students’ demographics including age, sex and marital status. Furthermore, their parents’ educational level was asked, which we regrouped into higher education (higher secondary or vocational school or university), and lower education (intermediate secondary or vocational school, lower secondary or vocational school or primary school). We also asked for parents’ current working hours and dichotomized full-time or part-time work.

Then, students were asked to choose their favourite speciality from a list of seven specialities (internal medicine, psychiatry, neurology, paediatrics, surgery, gynaecology and family medicine) or the options ‘other, namely…’ or ‘I don’t know’. If a student gave more than one answer, we categorized this under ‘I don’t know’. The working hours students would prefer in the future were categorized as full-time or part-time preference, no paid work or ‘I don’t know’. We created a dichotomous variable with a full-time or part-time preference to specify these working hour preferences. A part-time worker has been defined as an ‘employed person whose normal hours of work are less than those of a comparable full-time worker’ [26]. A doctor’s full-time working week is over 40 h. We defined part-time work as less than 36 h.

Students’ opinions about 11 issues on work-life balance, six on career issues, for example ‘The following reason contributes to my speciality choice: possibilities for reconciliation of work and care’, and five on care tasks, for example ‘Do you think that your job and career goals affect your choices regarding having a family?’ These work-life issues were collected and assessed with a 5-point Likert scale (totally disagree = 1 to totally agree = 5). We categorized each work-life issue variable into ‘disagree’ (including ‘totally disagree’, ‘disagree’, ‘neutral’) and ‘agree’ (including ‘agree’, ‘totally agree’), creating a dichotomous variable for further analysis.

### Analysis

We analyzed differences between male and female graduates in demographic variables, working hour preferences, work-life issues and speciality choices with Chi square tests (categorical variables) or unpaired *t* tests (continuous variables).

We used logistic regression modelling with the independent variables sex or a full-time or part-time preference to assess the relation of sex with speciality choice and of a full-time or part-time preference with speciality choice. In addition we modelled the relation of sex or a full-time or part-time preference with work-life issues.

We tested the mediating effect of work-life issues on the relations between sex and speciality choice or between a full-time or part-time preference and speciality choice using a method as proposed by Baron and Kenny [27]. Speciality preference was considered to be the dependent variable, work-life issues were the mediators, and sex or full time or part time were the independent variables. For mediation, three conditions had to be met: the independent variables had to be significantly related to the potential mediator, the mediator had to be significantly related to the dependent variable and the independent variables had to be significantly associated with the dependent variable. Mediation analysis was therefore only conducted were these relations became apparent in the preceding logistic analyses. Subsequently, the results of two separate regressions were compared; the dependent variable regressed on the independent variables, and the dependent variable regressed on the independent variables and the mediator. In order for mediation to be established, the odds ratios obtained from the latter model must be smaller than those from the first model. We assumed some form of mediation if the effect of work-life issues on speciality choice remained significant after controlling for sex or work-life issues. If sex or full-time or part-time preference were no longer significant after introducing work-life issues into the model, this finding supported full mediation; if the relation between sex or working hours and speciality choice remained significant partial mediation was supported.

In all tests the significance level was set on *p* < 0.05. For statistical analysis the software IBM SPSS statistics 20 was used.

## Results

### Demographics

A total of 1,050 graduates, of whom 73.3 % women, responded to a questionnaire on Gender Issues in Medicine at the end of their study (response rate 83 %). The male-to-female ratio was comparable in all 4 years of the cohort.

The mean age of women graduates was 24.4 years and that of men 24.9 years (Table 1). Approximately two-thirds of all students were in a relationship and 2 % of the students had children. The educational level and full-time or part-time employment of graduates’ parents did not differ among female and male students. Two-thirds of the students’ fathers and half of the students’ mothers were highly educated. Almost all of these fathers and not even one-quarter of the mothers worked full-time.

**Table 1:** Demographics of study population

	Female % (*n*)	Male *%* (*n*)	*p*
Age: Mean (SD; Min–Max)	24.4 (2.4; 21–46)	24.9 (3.1; 21–45)	0.015*
Civil status			0.272
Single	37.0 (286)	33.3 (91)	
In a relationship	63.0 (486)	66.7 (182)	
Children			0.033*
Yes	1.3 (10)	3.3 (9)	
No	98.7 (752)	96.7 (260)	
Mother’s education			0.189
No/lower	48.5 (370)	53.1 (144)	
Higher	51.5 (393)	46.9 (127)	
Father’s education			0.495
No/lower	36.0 (273)	33.7 (91)	
Higher	64.0 (485)	66.3 (179)	
Mother’s work			0.278
Full-time	23.3 (133)	27.1 (55)	
Part-time	76.7 (438)	72.9 (148)	
Father’s work			0.677
Full-time	87.3 (542)	86.2 (187)	
Part-time	12.7 (79)	13.8 (30)	

* *p* < 0.05

## Influence of sex

Sex was of influence in a choice for surgery which was more often preferred by male graduates, and family medicine which was more often preferred by female graduates (Table 2).

**Table 2:** The influence of sex or working hour preference on speciality choice

	Female	Male	Influence of sex		Influence of working hours	
	% (*n*)	% (*n*)	OR (95 % CI)	*p*	OR (95 % CI)	*p*
Specialities						
Internal medicine	12.3 (95)	14.9 (41)	0.78 (0.54/1.18)	0.261	2.01 (1.35/3.0)	0.001*
Psychiatry	1.9 (15)	2.5 (7)	0.76 (0.31/1.87)	0.545	0.41 (0.17/1.0)	0.047*
Neurology	3.7 (29)	4.7 (13)	0.78 (0.40/1.53)	0.475	2.05 (1.02/4.14)	0.045*
Paediatrics	5.5 (43)	4.0 (11)	1.41 (0.72/2.78)	0.320	0.84 (0.49/1.46)	0.541
Surgery	7.6 (59)	21.5 (59)	0.30 (0.20/0.45)	0.000*	4.98 (2.93/8.45)	0.000*
Gynaecology	7.1 (55)	4.7 (13)	1.54 (0.83/2.86)	0.173	1.47 (0.88/2.47)	0.146
Family medicine	32.5 (252)	18.5 (51)	2.12 (1.51/2.97)	0.000*	0.33 (0.25/0.44)	0.000*
Other	13.7 (106)	15.3 (42)	0.88 (0.60/1.30)	0.514	1.15 (0.81/1.65)	0.433
I don’t know	15.6 (121)	13.8 (38)	1.15 (0.78/1.71)	0.476	0.91 (0.65/1.28)	0.579

Legend: Graduates’ speciality consideration (outcome): modelling the probability of choosing a speciality preference (not choosing it = ref.), Independent variables: either sex (female, male = ref.) or working hours (full-time work, part-time work = ref.)

*OR* Odds ratio, 95 % *CI* confidence interval

* *p* < 0.05

The influence of sex was present in almost all work-life issues (Table 3). Male students, more often than female students, anticipated that their partner would be less ambitious than themselves. Furthermore, men more often stipulated that their partner’s career would affect family and that having a family would negatively influence their partner’s career. Likewise, women indicated more often than men that indeed their career would affect family life. Furthermore, female students, to a higher degree than male students, emphasized equality in childcare and in household chores and stipulated a wish to outsource childcare.

**Table 3:** The influence of sex or working hour preference on work-life issues

		Female	Male	Influence sex		Influence working hours	
		% (*n*)	% (*n*)	OR (95 % CI)	*p*	OR (95 % CI)	*p*
	*Work-life issues*						
	Career						
1	Same opportunities concerning career as partner	41.1 (314)	39.1 (106)	1.14 (0.87–0.34)	0.340	1.34 (1.04–1.72)	0.023*
2	Partner less ambitious than you	37.1 (284)	44.5 (121)	0.65 (0.49–0.00)	0.004*	1.74 (1.36–2.24)	0.000*
3	Job and career goals affect choice of having a family	76.9 (587)	64.6 (175)	1.93 (1.33–0.00)	0.001*	0.94 (0.65–1.35)	0.742
4	Having a family affects job and career aspirations	64.4 (487)	62.9 (171)	1.01 (0.72–0.97)	0.970	0.96 (0.70–1.30)	0.777
5	Job and career goals of partner affect choice of having a family	48.6 (370)	61.3 (166)	0.53 (0.37–0.00)	0.001*	1.43 (1.08–1.91)	0.014*
6	Having a family affects job and career aspirations of partner	49.1 (372)	64.3 (175)	0.51 (0.35–0.00)	0.000*	1.01 (0.75–1.36)	0.931
	Care						
7	Equally share household chores	72.9 (557)	57.3 (157)	2.11 (1.53–0.00)	0.000*	0.77 (0.57–1.06)	0.104
8	Household chores by someone else^1^	60.8 (465)	50.6 (137)	1.42(1.05–0.02)	0.024*	1.05 (0.79–1.39)	0.751
9	Equal care of the children	60.9 (465)	50.5 (138)	1.37 (0.99–0.06)	0.060	1.11 (0.82–1.51)	0.485
10	Taking care of the children by day care centre	71.0 (540)	59.0 (161)	1.59 (1.09–0.02)	0.016*	1.12 (0.78–1.60)	0.553
11	Taking care of the children by nanny and grandparents	76.9 (586)	59.6 (162)	2.02 (1.38–0.00)	0.000*	0.94 (0.65–1.36)	0.754

Legend: Graduates’ work-life issue consideration (outcome): modelling the probability of agreement to it (agreement means choosing value 4–5, not agreeing is value 1–3 = ref.), Independent variables: either sex (female, male = ref.) or working hours (full-time work, part-time work = ref.)

*OR* Odds ratio, 95 % *CI* confidence interval

* *p* < 0.05

Indecisive students hesitated mainly between two specialities (F 55.4 %/n = 67 vs M 60 %/n = 24). This seems unaffected by their sex and views on working hours.

### Influence of working hour preference

Male students highly preferred full-time work (full-time 84 %/n = 231, part-time 15.3 %/n = 42; *p* = 0.000), whereas female students showed an interest in both (full-time 47.4 %/n = 368; part-time F 51.2 %/n = 397; *p* = 0.000).

A full-time preference was highly related to a choice for surgery and to a choice for internal medicine or neurology (Table 2). A part-time preference increased the likelihood of the student choosing family medicine or psychiatry.

A full-time or part-time preference was not a major influential factor in work-life issues. A full-time preference was associated with equality in career opportunities between partners, with the expectation that the partner would be less ambitious or that the career of the partner would affect choices of having a family.

### Influence of work-life issues

Work-life issues influenced the choice for some specialities to a higher degree than others.

Issues relating to career matters influence the choice for surgery and family medicine. If students anticipated that their partner would be less ambitious, this elevated the likelihood of them choosing surgery (*p* = 0.004, OR = 1.87; CI 1.23–2.58) and lowered the chance of them choosing family medicine (*p* = 0.001, OR = 0.64; CI 0.49–0.84). Students who anticipated that their career would influence their family life were less likely to prefer surgery (*p* = 0.021, OR = 0.56; CI 0.35–0.93). If the students anticipated that their partner’s career would influence family life (*p* = 0.017, OR = 0.69; CI 0.51–0.94) or that family life would affect their partner’s career (*p* = 0.033, OR = 0.56; CI 0.33–1.00) this lowered their choice for family medicine.

Work-life issues relating to care matters influenced choices for surgery, gynaecology, family medicine, internal medicine and psychiatry. Agreement on sharing household chores reduced the likelihood of choosing surgery (*p* = 0.002, OR = 0.51; CI 0.33–0.77), or the category other specialities (*p* = 0.045, OR = 0.66, CI 0.44–0.99) and increased the preference for family medicine (*p* = 0.001, OR = 1.83; CI 1.27–2.56). Equal care for children reduced the chance that students would have a preference for internal medicine (*p* = 0.048, OR = 0.66; CI 0.43–1.00). Agreement on childcare by day care (*p* = 0.17 OR = 11.23; CI 1.55–81.57) or childcare by a nanny (*p* = 0.042, OR = 3.37; CI 1.04–10.86) highly enhanced the likelihood of a choice for gynaecology. If students anticipated childcare by a nanny, this meant they were less likely to choose psychiatry (*p* = 0.011; OR = 0.31; CI 0.12–0.76).

### Mediation by work-life issues

Although we found partial mediation for two work-life issues (expectation of partner being less ambitious than you and equally sharing household chores) on the relation between sex and the choice of surgery and family medicine and partial mediation of the expectation of partner being less ambitious than you on the relation between full-time preference, no substantial changes in odds ratios were found. Therefore, a mediating effect of work-life issues on the relation of sex and full-time preference on the choice of speciality is limited. There is a direct relation between sex and full-time/part-time preference and speciality choice.

## Discussion

Amongst graduating medical students, women formed the majority of our study population and these female students are far less interested in full-time work than male students. A full-time or part-time work focus appears highly influential in speciality choice-making. New to the study is the finding that preferences for working full-time or part-time work are decisive for speciality choice whereas the content of a speciality, which is generally assumed to be the most important influencing factor, may not be the main decisive factor. Moreover, male or female gender has a large influence on work-life issues. We found that both male and female students anticipate the influence of the women’s career on family life. In this matter, men foremost anticipate that their partner is less ambitious, whereas women emphasize equality in care tasks. In addition, a full-time preference is more often associated with agreement to equality in career opportunities between partners or the expectation that their partner will be less ambitious. When students assume that their partner will be less ambitious, this increases the prevalence of a choice for surgery and decreases a choice for family medicine. The above suggests that the way in which male or female graduating students consider their own ambitions, as well as their partners’, or anticipate equality in care responsibilities, plays a significant role in their speciality choice-making.

It seems that the influence of sex on speciality choice is limited. Being male only significantly relates to a choice for surgery and more female students opt for family medicine. Notwithstanding, female gender does, to a high extent, influence the working hour preference. Moreover, our findings show that full-time or part-time preference is related to specific speciality choices. For instance, we found an association between a full-time work focus and the choice for surgery or gynaecology and a part-time focus was related to a choice for family medicine. Reasons mentioned by women that deter them from surgical training are the length of the training to become a specialist, competition, a lack of female role models and a perceived negative attitude of surgeons towards female physicians [28–30]. In contrast, gynaecology is popular with women, which could be considered to be a comparable speciality to surgery concerning workload [20]. Sex relates to working hour preferences but does fully explain differences in distribution of men and women across specialities.

Our study also shows that sex to a high extent relates to work-life issues on career and care matters. To our knowledge our study is the first to explicitly test the mediation of work-life issues in speciality choice making. Expecting to have a less ambitious partner was related to a higher preference for surgery among women. Both men and women estimate a lower career opportunity for women. There are subtle conflicting differences as men expect their partners to be less ambitious whereas women more often expect equality in care tasks. Our findings suggest that gender is important in speciality choice making, through particular expectations and beliefs about work-life issues.

### Limitations

Our results are based on a cohort of 1,050 graduating medical students and we had a high response rate. Nevertheless, our study has some limitations. We cannot rule out the possibility that the period on which the participants completed the questionnaire could have biased the outcome of the speciality preferences in our study. For example, a choice for family medicine could relate to participants just ending their general practice clerkship [31]. However, after ending the clerkship the result of the motivating effect will most likely disappear by the time they graduate [32]. Furthermore, we tested mediation with a four-step regression analysis. A potential problem is that with this approach we missed some true mediating effects (Type II errors), as we do not really test the significance of the indirect pathway but analyze a compound pathway through work-life issues [27].

### Implications

The possibility for physicians who are parents to work full-time and at the same time be satisfied with their children’s daily life cannot be seen as a private issue. Specialities should become more active in implementing policies that target underlying norms and make a cultural change. Also, they may develop practical solutions in the organization of work in a department as such enhancing the attractiveness of certain disciplines. In order to prevent a loss of female physicians, the utility of both men and women in a profession can be organized by providing affordable and excellent childcare support: for instance, day care centres closer to the hospitals for staff. Medical education should provide a framework which consciously and actively participates in the professional career choices of students and issues related to work and care. Such coaching will give both male and female students the opportunity to make a well-informed career choice.

## Conclusion

Female graduates far less often than men prefer full-time work and over two-thirds of our study population are female. Preferred working hours were highly influential in speciality choice making, as we demonstrated that a full-time preference relates to a choice for surgery and a part-time preference more often leads to a choice for family medicine. More male graduates chose surgery and more female graduates family medicine. Both male and female students anticipate the influence of the women’s career on family life, meaning that men foremost anticipate that their partner is less ambitious, whereas women emphasize equality in care tasks. These work-life issues affect the influence of sex and working hour preference on speciality choices, as is illustrated by female students who more often prefer surgery when they expect that their partner will be less ambitious. The way male and female medical graduates consider career and responsibilities in caring roles plays a role in their choice making. Combining work and childcare cannot be seen as a private issue and action on a structural level by politicians and health care planners seems a necessity. Medical education should offer coaching in the professional career choices of students and issues related to work and care.

## Essentials

- Female graduates prefer full-time work far less than male graduates.
- A full-time or part-time preference relates to specific speciality choices.
- Both male and female students anticipate that foremost the career of women will be negatively influenced by family life.
- A full-time preference relates to work-life issues as equality in career opportunities or having a less ambitious partner and as such influences speciality choice.
